# Modelling the depth‐dependent VASO and BOLD responses in human primary visual cortex

**DOI:** 10.1002/hbm.26094

**Published:** 2022-10-03

**Authors:** Atena Akbari, Saskia Bollmann, Tonima S. Ali, Markus Barth

**Affiliations:** ^1^ Centre for Advanced Imaging University of Queensland Brisbane Australia; ^2^ ARC Training Centre for Innovation in Biomedical Imaging Technology The University of Queensland Brisbane Australia; ^3^ School of Information Technology and Electrical Engineering The University of Queensland Brisbane Queensland Australia

**Keywords:** blood‐oxygenation‐level‐dependent, cerebral blood volume, cortical layers, depth‐dependent, laminar fMRI, primary visual cortex, VAscular‐Space‐Occupancy

## Abstract

Functional magnetic resonance imaging (fMRI) using a blood‐oxygenation‐level‐dependent (BOLD) contrast is a common method for studying human brain function noninvasively. Gradient‐echo (GRE) BOLD is highly sensitive to the blood oxygenation change in blood vessels; however, the spatial signal specificity can be degraded due to signal leakage from activated lower layers to superficial layers in depth‐dependent (also called laminar or layer‐specific) fMRI. Alternatively, physiological variables such as cerebral blood volume using the VAscular‐Space‐Occupancy (VASO) contrast have shown higher spatial specificity compared to BOLD. To better understand the physiological mechanisms such as blood volume and oxygenation changes and to interpret the measured depth‐dependent responses, models are needed which reflect vascular properties at this scale. For this purpose, we extended and modified the “cortical vascular model” previously developed to predict layer‐specific BOLD signal changes in human primary visual cortex to also predict a layer‐specific VASO response. To evaluate the model, we compared the predictions with experimental results of simultaneous VASO and BOLD measurements in a group of healthy participants. Fitting the model to our experimental data provided an estimate of CBV change in different vascular compartments upon neural activity. We found that stimulus‐evoked CBV change mainly occurs in small arterioles, capillaries, and intracortical arteries and that the contribution from venules and ICVs is smaller. Our results confirm that VASO is less susceptible to large vessel effects compared to BOLD, as blood volume changes in intracortical arteries did not substantially affect the resulting depth‐dependent VASO profiles, whereas depth‐dependent BOLD profiles showed a bias towards signal contributions from intracortical veins.

## Graphical abstract

In this study, we have measured the depth‐dependent blood‐oxygenation‐level‐dependent and VAscular‐Space‐Occupancy profiles in the human primary visual cortex and then simulated these responses using a simple cortical vascular model to obtain an estimate of CBV change in different vascular compartments upon neural activity.

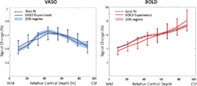

## 1 INTRODUCTION

High‐resolution functional magnetic resonance imaging (fMRI) offers the potential to measure depth‐dependent hemodynamic responses, which can provide insights into cortical information processing and microcircuits of the human brain (Douglas & Martin, 2004; Lawrence et al., 2019; Stephan et al., 2019). Numerous studies have investigated the function of cortical layers using the blood‐oxygenation‐level‐dependent (BOLD) contrast (Ogawa et al., 1990) in animals and humans (Aitken et al., 2020; Bollmann & Barth, 2020; Chen et al., 2013; de Hollander et al., 2021; Goense et al., 2012; Goense & Logothetis, 2006; Koopmans et al., 2010; Polimeni et al., 2010; Poplawsky et al., 2015; Ress et al., 2007; Self et al., 2017; Silva & Koretsky, 2002; van Dijk et al., 2020; Vizioli et al., 2020; Yu et al., 2014; Zaretskaya et al., 2020); for a brief history of the field see also Norris and Polimeni (2019). Despite the high sensitivity of this technique, it suffers from limited specificity due to signal leakage in draining veins carrying blood from (activated) lower layers to superficial layers and further to the pial veins (Duvernoy et al., 1981; Kim et al., 1994; Turner, 2002). This low specificity was the motivation to develop non‐BOLD contrast mechanisms, such as cerebral‐blood‐volume (CBV) imaging, which is expected to be predominantly sensitive to hemodynamic responses in the microvasculature (Gagnon et al., 2015; Jin & Kim, 2006, 2008a; Kim & Kim, 2010, 2011a; Poplawsky et al., 2015; Silva et al., 2007; Vanzetta et al., 2005; Zhao et al., 2006).

A noninvasive method for CBV imaging is vascular‐space‐occupancy (VASO) (Lu et al., 2003), which takes advantage of the difference in blood and tissue $T_{1}$ to image the tissue signal while the blood signal is nulled (Huber, Ivanov, et al., 2014; Lu et al., 2003). Since the development of this contrast and its translation to 7 Tesla (T), several studies in animals and humans have been conducted in the areas of method development (Beckett et al., 2020; Chai et al., 2019; Huber et al., 2015; Huber et al., 2016; Yu et al., 2014), analysis strategies (Huber et al., 2021; Polimeni et al., 2018), and applications to cognitive neuroscience (Finn et al., 2019; Huber, Goense, et al., 2014; Huber, Handwerker, et al., 2017; Kashyap et al., 2018; Oliveira et al., 2021; van Kerkoerle et al., 2017). However, to interpret the experimental results and account for both neural and vascular contributions to the fMRI signal, detailed models are required (Buxton et al., 2004). Several studies have modelled the BOLD response for both low‐ and high‐resolution acquisitions (Baez‐Yanez et al., 2020; Buxton et al., 1998; Buxton et al., 2004; Gagnon et al., 2015; Havlicek & Uludağ, 2020; Heinzle et al., 2016; Markuerkiaga et al., 2016; Uludağ et al., 2009). Recently, Genois et al. (2020) modelled BOLD and VASO signals using a vascular anatomical network (VAN) model (Boas et al., 2008) of the rat brain to investigate intra and extravascular contributions to the BOLD signal and BOLD contribution to the VASO signal. However, no simulations of depth‐dependent BOLD or VASO signals were provided in this study. Given the potential of VASO imaging for layer fMRI, we set out to model the depth‐dependent VASO signal changes in human primary visual cortex (V1) employing a detailed model of the underlying macro‐ and micro‐vasculature (Markuerkiaga et al., 2016). In this work, we extended and modified the “cortical vascular model” (Markuerkiaga et al., 2016) to simulate VASO responses in addition to BOLD responses at the laminar level. This model is based on histological observations in macaque primary visual cortex and considers various vascular features, such as vessel diameter, length, density, and distribution to simulate intra and extravascular BOLD and VASO signals across cortical layers. We added intracortical (diving) arteries (ICAs) to the modelled region, as it is hypothesized that these play a role in the functional VASO response based on previous observations (Gagnon et al., 2015; Vanzetta et al., 2005). We also modified the artery to vein ratio defined in the model such that it is applicable to the human brain (Cassot et al., 2009; Schmid et al., 2019). To fit the predictions of the now extended model to experimental data, we performed simultaneous BOLD and VASO imaging in a group of healthy participants with sub‐millimetre resolution at 7 T. The model fitting then provided estimates of CBV and oxygenation changes in micro‐vascular (arterioles, capillaries, venules) and macrovascular (ICAs and intracortical veins [ICVs]) compartments at each cortical depth. Furthermore, we investigated the sensitivity of both VASO and BOLD contrasts to changes in the underlying physiological parameters, that is, CBV and oxygenation.

To the best of our knowledge, this is the first study reporting depth‐dependent experimental VASO profiles in the human primary visual cortex and the first depth‐dependent VASO simulation study investigating the underlying physiological mechanism, such as the effect of baseline CBV on the resulting depth‐dependent BOLD and VASO profiles. In the following sections, we briefly summarize the general structure of the previously developed cortical vascular model (2.1 and 2.2), and then describe the applied changes to simulate the VASO and BOLD responses (2.3 and 2.4).

## 2 THEORY AND SIMULATIONS

### 2.1 The cortical vascular model

**FIGURE 1:**
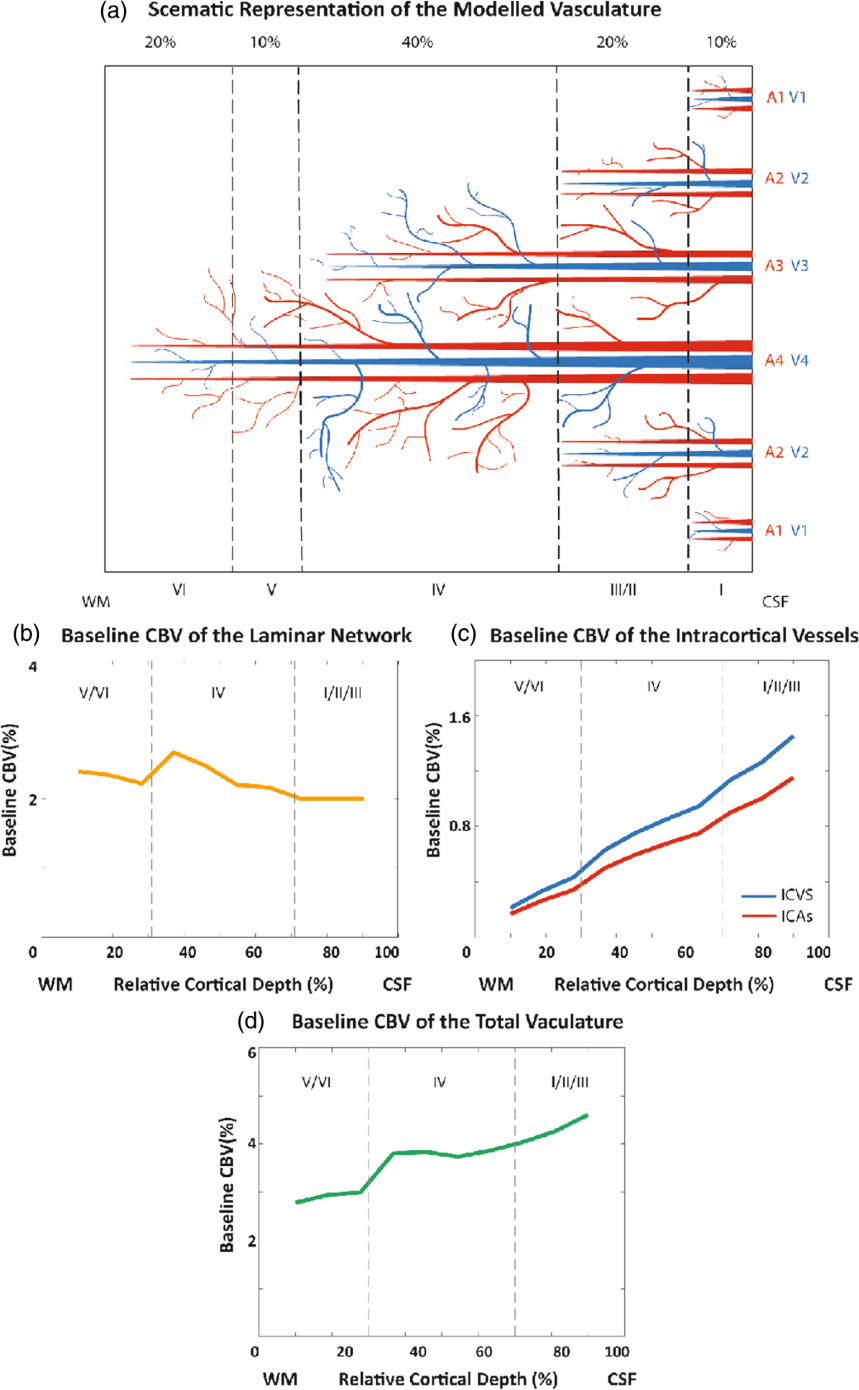
Vascular model of intracortical vessels and baseline cerebral blood volume of the different vascular compartments. (a) Schematic of the vascular features of the primary visual cortex illustrating the 2–1 artery‐to‐vein ratio. (b) Baseline blood volume of the laminar network (i.e., arterioles, capillaries, and venules) as a function of cortical depth following Weber et al. (2008). (c) Estimated baseline blood volume of the intracortical vessels (ICAs and ICVs). (d) Estimated baseline blood volume of the total vasculature.

The cortical vascular model developed by Markuerkiaga et al. (2016) simulates the steady‐state BOLD response in a depth‐dependent manner in human primary visual cortex. The model divides the brain vasculature into two groups: (i) the microvasculature forming a network of tangled, randomly oriented arterioles, capillaries, and venules called the *laminar network*, where the vessel distribution and blood volume varies as a function of depth. (ii) the macrovasculature in the form of intracortical veins (ICVs) that drain the microvasculature towards the cortical surface. In the original version of the cortical vascular model (Markuerkiaga et al., 2016), the vessel distribution in the laminar network was 21% arterioles, 36% capillaries, and 43% venules. However, these values are based on the rat brain (Boas et al., 2008), and the artery‐to‐vein ratio should be reversed for the human brain (Baez‐Yanez et al., 2020; Cassot et al., 2009; Schmid et al., 2019). Thus, we assumed the following vessel density in the laminar network: 43% arterioles, 36% capillaries, and 21% venules. The model investigates the effect of the ascending veins on the BOLD signal by calculating the diameter, blood velocity, and mass flux of the ICVs in each layer. To simulate the VASO response, we added diving arteries to the modelled region, as several studies have shown that dilation mainly occurs in arteries and arterioles (Gagnon et al., 2015; Kim & Kim, 2011a; Vanzetta et al., 2005). To do so, a vascular unit centered on two adjacent principal veins (V3 and V4), surrounded by an arterial ring is modelled in this work with an artery‐to‐vein ratio of 2‐to‐1 (Cassot et al., 2009; Lauwers et al., 2008; Schmid et al., 2019). The diameter of this vascular unit is around 0.75–1 mm. Considering the 2.5 mm thickness of V1 (Fischl & Dale, 2000), we simulated 10 voxels with the size of 0.75 × 0.75 × 0.25 mm^3^, that is, signal change of one venous unit at 10 cortical depths. Figure 1a shows a schematic of the modelled intracortical arteries and veins following Duvernoy et al. (1981) in which vessels are categorized based on their diameter and penetration depth.

The diameter of the intracortical vessels at each depth is calculated following the steps described in Markuerkiaga et al. (2016). In brief, based on the mass conservation law, the incoming mass flux $\left(p\right)$ to the arteries should be equal to the outgoing flux from the veins in the modelled region at steady state. Similarly, the mass flux from each depth is the mass flux from within the microvasculature plus the mass flux in the macrovasculature from the previous layer. In general, the mass flux through vessels can be calculated as:

(1) $$p=r^{2}\cdot v,$$

where r is the vessel radius and v is the blood velocity. Assuming a linear relationship between vessel diameter and blood velocity v expressed in the parameter α, we can rewrite this as:

(2) $$p=\frac{d^{3}}{4}\cdot \alpha ,$$

(3) $$\alpha =\frac{v}{d},$$

The mass flux p through a single capillary is calculated assuming a capillary diameter of d=8μm and v=1.6mm/s (Boas et al., 2008; Zweifach & Lipowsky, 1977). Then, p through ICVs and ICAs present in each layer is calculated starting from the layer closest to the white‐matter (WM) border based on the number of capillaries in that layer:

(4) $$p_{\text{ICVs}}=\frac{N_{\mathrm{cap}}}{N_{\text{ICVs}}}\cdot p_{\mathrm{cap}}.$$

For the rest of the layers, p in each layer is the summation of the mass flux within that layer and the mass flux from the previous layer(s). The same calculation for ICVs applies for ICAs, but with twice the number of arteries (Schmid et al., 2019). Note that for this steady‐state model, the direction of flow does not play a role when calculating the mass flux at each depth. The only relevant considerations are the mass conversation law, that is, mass flux entering a layer should be equal to the mass flux that exits this layer. Thus, the mass flux in each layer is equal to the additional mass flux ($p_{\mathrm{cap}}$) from the laminar network in that layer plus the mass flux in the preceding layers. Therefore, the calculation can be done in both bottom‐up or top‐down directions. The α values in pre and postcapillaries compartments were calculated assuming d=12.5μm and v=2mm/s in postcapillary and v=4mm/s in the precapillary segment of the vasculature (Equation 3) (Zweifach & Lipowsky, 1977). Then, based on the mass flux for each vessel at each layer, the vessel diameter and the blood velocity of the macrovasculature can be calculated using Equation 2 and 3. Table 1 shows the estimated diameters of intracortical arteries and veins, which are in line with the values reported in Duvernoy et al. (1981).

**TABLE 1:** The average diameter (in μm) of intracortical arteries and veins in the modelled vascular unit centred on two intermediate‐sized veins (V3 and V4) and surrounded by four intermediate‐sized arteries (two A3 and two A4).

The average diameter (μm) of the Intracortical vessels (ICAs and ICVs)								
Vessel type	V4	V3	V2	V1	A4	A3	A2	A1
Layer I	69	53	32	20	43	33	20	13
Layer II/III	67	51	26		43	32	16	
Layer IV	62	41			39	26		
Layer V	56				35			
Layer VI	44				28			

*Note*: For reference, the ICV diameters of group 1–4 reported in Duvernoy et al. (1981) range from 20 to 65 μm, and the diameter of the corresponding ICAs range from 10 to 40 μm.

The baseline blood volume of the laminar network taken from Weber et al. (2008) was interpolated to the number of voxels being simulated, resulting in 2%–2.7% baseline CBV (Figure 1b). The intracortical baseline CBV is calculated as:

(5) $$\mathrm{CB}V_{\text{base}}^{\text{ICAs},\text{ICVs}}=\pi \frac{d^{2}}{4}/l^{2},$$

in which l is the simulated voxel length (0.75 mm) yielding a baseline CBV in ICVs ranging from 0.2%–1.4% and a baseline CBV in ICAs ranging from 0.2% to 1.2% (Figure 1c). The average of the total baseline CBV of the modelled vasculature is 3.7% (Figure 1d). This vascular model is then combined with the MR signal model (see section 2.2) to calculate the layer‐dependent signal changes.

### 2.2 BOLD and VASO MR signal models

The MR signal model employed here is a steady state model contrasting signal levels at baseline and during activity (Markuerkiaga et al., 2016; Uludağ et al., 2009). At baseline, the total MR signal $S_{\mathrm{tot}}^{\text{base}}$ is the sum of the intra (IV) and extravascular (EV) signal components (Buxton, 2009; Obata et al., 2004; Uludağ et al., 2009):

(6) $$S_{\text{base}}^{\mathrm{tot}}=\left(1-{\mathrm{CBV}}_{\text{base}}\right)\cdot S_{\text{base}}^{\mathrm{EV}}+\sum_{i}S_{\text{base},i}^{\mathrm{IV}}\cdot \mathrm{CB}V_{\text{base},i},$$

where CBV is the baseline blood volume, and i denotes different vascular compartments, that is, arterioles, capillaries, venules, ICVs, and ICAs. In the following, we describe the intra and extravascular BOLD and VASO signals when using a GRE readout at 7 T.

The BOLD signal is approximated as a mono‐exponential decay (Yablonskiy & Haacke, 1994), where TE is the echo time, $S_{0}$ the effective spin density at TE=0, and $R_{2}^{*}$ the transverse relaxation rate:

(7) $$S^{\text{BOLD}}=S_{0}\cdot e^{-T_{E}\cdot R_{2}^{*}}.$$

The transverse relaxation rate is the sum of the intrinsic ($R_{2,0}^{*}$) and hemoglobin (Hb)‐induced transverse relaxation rates ($R_{2,\mathrm{Hb}}^{*}$):

(8) $$R_{2}^{*}=R_{2,0}^{*}+R_{2,\mathrm{Hb}}^{*}.$$

All intrinsic and Hb‐induced $R_{2}^{*}$ values used in this model (Blockley et al., 2008; Uludağ et al., 2009) are summarized in Table 2. Extra and intravascular BOLD signals are estimated using their corresponding relaxation rates. In short, the Hb‐induced extravascular relaxation rate is calculated according to the susceptibility‐induced shift at the surface of the vessel depending on the oxygenation level $\;\left(Y\right)$ (Uludağ et al., 2009). The intravascular $T_{2}^{*}\;$ of the ICVs (intrinsic and Hb‐induced) are very short at high field (7 T and above). Therefore, the intravascular signal in veins approaches zero (Uludağ et al., 2009), and the main intravascular contribution comes from the arterial and capillary side of the vasculature.

**TABLE 2:** The intrinsic and Hb‐induced intra and extravascular transverse relaxation rates used in the BOLD signal model for 7T (Uludağ et al., 2009).

Compartment	Intrinsic relaxation time	Hb‐induced relaxation time
Intravascular (blood)	$R_{2,\mathrm{IV},0}^{*}$(s^−1^)	$R_{2,\mathrm{IV},\mathrm{Hb}}^{*}$(s^−1^)
	67	$C\cdot {\left(1-Y\right)}^{2}$
Extravascular (tissue)	$R_{2,\mathrm{EV},0}^{*}$ (s^−1^)	$R_{2,\mathrm{EV},\mathrm{Hb}}^{*}$ (s^−1^)
	34	$R_{2,\mathrm{EV},\mathrm{Hb}}^{*}=\left(e\cdot {\Delta \nu }_{s}+f\right)\cdot {\mathrm{CBV}}_{i}$ ${\Delta \nu }_{s}=\frac{∆\chi_{0}}{4\pi }\cdot \mathrm{Hct}\cdot \left(\left|Y_{\mathrm{off}}-Y\right|\right)\cdot \gamma \cdot B_{0}$

*Note*: C, constant that depends on the magnetic field strength, that is, 7 T (= 536.48). ${\Delta \nu }_{s}$, the susceptibility‐induced shift at the surface of the vessel corresponds to Larmor frequency shift (depends on Y). $∆\chi_{0}$, the susceptibility of blood with fully deoxygenated blood (=4π·0.264ppm). $Y_{\mathrm{off}}$, the oxygenation level that produces no magnetic susceptibility difference between intravascular and extravascular fluids (= 95%). Hct=40%. e=0.0453andf=−0.19: fitting coefficients.

Following neural activity and changes in blood volume and oxygenation, the total MRI signal is:

(9) $$S_{\mathrm{act}}^{\mathrm{tot}}=\left(1-{\mathrm{CBV}}_{\mathrm{act}}\right)\cdot S_{\mathrm{act}}^{\mathrm{EV}}+\sum_{i}S_{\mathrm{act}}^{\mathrm{IV}}\cdot \mathrm{CB}V_{\mathrm{act},i}.$$

where ${\mathrm{CBV}}_{\mathrm{act}}={\mathrm{CBV}}_{\text{base}}+\mathrm{\Delta CB}V_{\mathrm{abs}}$ and $\mathrm{\Delta CB}V_{\mathrm{abs}}=\mathrm{\Delta CB}V_{\mathrm{rel}}\cdot \mathrm{CB}V_{\text{base}}$. Note that $\mathrm{\Delta CB}V_{\mathrm{abs}}$ denotes the blood volume change upon activation in ml/100 ml tissue commonly used (Huber et al., 2015; Lu et al., 2013) and $\mathrm{\Delta CB}V_{\mathrm{rel}}$ in % baseline blood volume, which is the definition implemented in the cortical vascular model. The increase in oxygenation is reflected in the shortening of the relaxation rates, which leads to increased extra and intravascular signal levels. The BOLD signal change in percent (%) following neural activity can be described as the signal difference between baseline and activation, normalized to the baseline signal level:

(10) $$\frac{\mathrm{\Delta S}}{S_{\text{base}}}=\frac{S_{\mathrm{act}}^{\mathrm{tot}}-S_{\text{base}}^{\mathrm{tot}}}{S_{\text{base}}^{\mathrm{tot}}}\cdot 100\;\left[\%\right].$$

For VASO, assuming a perfect inversion pulse, signal change arises only from the extravascular component, as the intravascular signal is nulled with an inversion pulse. The steady state nulled tissue signal is (Huber, Ivanov, et al., 2014; Lu et al., 2003; Lu et al., 2013):

(11) $$S^{\mathrm{EV},\text{nulled}}=S_{0}\left(1-\left(1+\varepsilon \right)e^{\frac{-T_{I}}{T_{1}}}+\varepsilon e^{\frac{-T_{R}}{T_{1}}}\right)\cdot e^{-\mathrm{TE}\cdot R_{2,\mathrm{EV}}^{*}},$$

in which ε is the inversion efficiency (here assumed to be equal to one), $T_{I}/T_{1}/T_{R}$ are the blood nulling time, longitudinal relaxation time, and repetition time, respectively. At the time of the blood nulling, a BOLD signal contamination—the $T_{2}^{*}-$ dependency—is still present and needs to be corrected. The dynamic division approach proposed by Huber, Ivanov, et al. (2014) removes the $T_{2}^{*}$‐contribution from the VASO signal by dividing the “nulled” by the “non‐nulled” signal, assuming equal extravascular BOLD contributions in both images and negligible intravascular BOLD signal^1^:

(12) $$S^{\mathrm{EV},\text{VASO}}=\frac{S^{\mathrm{EV},\text{nulled}}}{S^{\mathrm{EV},\text{non}-\text{nulled}}}=\left(1-\left(1+\varepsilon \right)\cdot e^{\frac{T_{I}}{T_{1}}}+\varepsilon \cdot e^{\frac{-T_{R}}{T_{1}}}\right)$$

Then, the VASO signal during baseline, activity, and total VASO signal change can be derived from Equation 6, 9, and 10 by considering only the extravascular components:

$$S_{\text{base}}^{\mathrm{tot},\text{VASO}}=\left(1-{\mathrm{CBV}}_{\text{base}}\right)\cdot S^{\mathrm{EV},\text{VASO}}$$

$$S_{\mathrm{act}}^{\mathrm{tot},\text{VASO}}=\left(1-\mathrm{CB}V_{\mathrm{act}}\right)\cdot S^{\mathrm{EV},\text{VASO}}$$

(13) $$\frac{\Delta S^{\text{VASO}}}{S_{\text{base}}^{\text{VASO}}}=\frac{-\Delta \mathrm{CB}V_{\mathrm{abs}}}{1-{\mathrm{CBV}}_{\text{base}}}.$$

Thus, VASO signal changes are only a function of CBV change and baseline CBV, and independent of oxygenation changes.

### 2.3 Model assumptions and simulations

To simulate depth‐dependent BOLD and VASO signal changes, the cortical vascular model outlined in section 2.1 requires ΔCBV and oxygenation values at baseline and activity for each depth and vascular compartment. Note that ΔCBV here denotes ΔCBV_rel_, which is given in percent of the baseline CBV, that is, an increase of 100% means that CBV during activation is twice as large as during baseline. Further, oxygenation is given in percent oxygen saturation, with 100% oxygenation corresponding to fully oxygenated blood. The resulting BOLD and VASO profiles are presented in percent signal change following Equation 10 and 13.

To find the input values that best fit the empirical data (see Section 3.2) we simulated numerous profiles for a wide range of input parameters (Table 3), and then calculated the root‐mean‐squared‐error (RMSE) for each simulated profile with the experimental result. The minimum RMSE value indicates the highest similarity between simulated and measured depth‐dependent responses. To investigate the effect of input parameters on the resulting depth‐dependent profiles, we also plotted the range of profiles obtained with RMSEs that are 20% higher than the minimum RMSE and extracted the corresponding input values for ΔCBV and oxygenation. For reference, we have included the original values used in Markuerkiaga et al. (2016) in square brackets in Table 3.

**TABLE 3:** The range of the model parameters for simulating VASO and BOLD depth‐dependent responses.

Vascular compartments CBV and oxygenation					
	Arterioles	Capillaries	Venules	ICVs	ICAs
$Y_{\text{base}}$	95%	85%	60%–75%^a^	60%–75%^a^	95%
	[95%]	[85%]	[60%]	[60%]	—
$Y_{\mathrm{act}}$	100%	95%	75–90%^b^	75%–90%^b^	100%
	[100%]	[95%]	[70%]	[70%]	—
$∆{\mathrm{CBV}}_{\mathrm{mid}}$	0%–90%	0%–90%	0%–90%	0%–90%	0–90%
	[16.6%]	[16.6%]	[16.6%]	[0%]	—

*Note*: The values in brackets refer to the values used in the original vascular model (Markuerkiaga et al., 2016). $\;Y_{\text{base}}\;$ and $\;Y_{\mathrm{act}}$ are the blood oxygenation at baseline and activation, and $∆{\mathrm{CBV}}_{\mathrm{mid}}$ refers to the CBV change in the middle layer. In the laminar network, the CBV change in middle layers is 1.5 times higher than in deep and superficial layers. In ICAs and ICVs, ΔCBV in middle and upper layers is 1.5 times higher than the change in deep layers.

^a^ Corresponds to $\;R_{2,\mathrm{IV}}^{*}=100-153\;\sec^{-1}.$

^b^ Corresponds to ${\;R}_{2,\mathrm{IV}}^{*}=72-100\;\sec^{-1}.$

To account for partial volume effects across layers and provide a better comparison between simulated and measured depth‐dependent responses (Markuerkiaga et al., 2016), we applied a smoothing kernel (Koopmans et al., 2011) to the simulated profiles. The resulting zero‐padded edges of the laminar profiles were thus excluded from the RMSE estimation and all simulated profiles, and only the central eight data points contributed.

**FIGURE 2:**
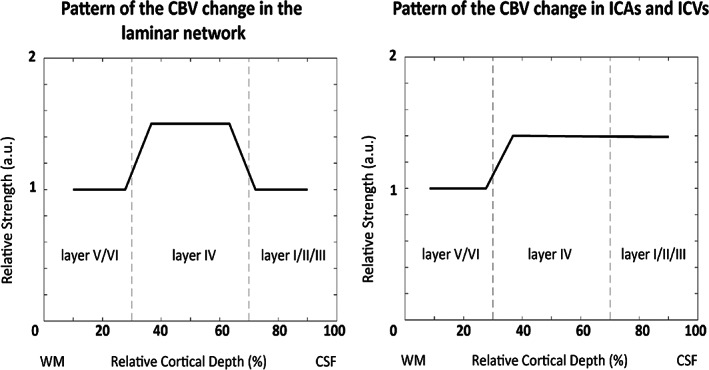
The pattern of the CBV change in the laminar network (left), and ICAs and ICVs (right) across the layers used in our simulations. According to the result shown in Figure 3 of Zhao et al. (2006), we assumed a higher CBV change in middle cortical layers by a factor of 1.5 in the laminar network. To accommodate this higher laminar CBV change we assumed a higher corresponding CBV change in ICAs and ICVs in the middle and superficial layers that drain the middle cortical layers. This step pattern represents an additional CBV change on top of the baseline CBV”.

In the original version of the cortical vascular model, Markuerkiaga et al. (2016) assumed the same increase in CBV of 16.6% (Griffeth et al., 2015) in each compartment of the laminar network across all depths. Using these input values, the resulting depth‐dependent VASO signal change (see Figure 8) did not exhibit the characteristic peak in the middle layers observed in our and other VASO experiments (Huber, Goense, et al., 2014). We therefore assumed a nonuniform activation strength across the layers. For the VASO simulations, we assumed in the laminar network a 1.5 times higher CBV increase in middle cortical layers (IV) compared with deep (V and VI) and superficial (I and II/III) layers (Zhao et al., 2006). In ICAs and ICVs, we assumed a correspondingly higher CBV change in middle and upper cortical layers compared with deep layers (see Figure 2):

(14) $$\mathrm{CB}V_{\mathrm{act},l}=\mathrm{CB}V_{\text{base},l}\cdot \left(1+\beta_{l}\cdot ∆\mathrm{CB}V_{\mathrm{rel}}\right),$$

in which β=1.5 for middle depths, and β=1 for superficial and deep cortical depths (Figure 2), and l denotes the cortical layers. We then simulated VASO profiles using this ratio for a range of ΔCBV values of 0%–90% for ICAs, arterioles, capillaries, venules, and ICVs (Table 3). In the original version of the cortical vascular model (Markuerkiaga et al., 2016), ICVs were considered not to dilate as shown by Hillman et al. (2007). However, other studies in cats (Kim & Kim, 2011b), mice (Takano et al., 2006), and humans (Chen & Pike, 2009; Stefanovic & Pike, 2005) observed dilation in ICVs, in particular for long stimulus duration. Therefore, we allowed this parameter to vary as well.

For the BOLD simulations, we used the ΔCBV values of the best fit from the VASO experiment, and instead varied oxygenation values between 60% and 75% at baseline and 75% and 90% at activation in the venules and ICVs (see Table 3). Following Markuerkiaga et al. (2016) and Uludağ et al. (2009), we assumed fixed oxygen saturation in ICAs, arterioles and capillaries at baseline and activation as outlined in Table 3, but varied the oxygen saturation of the venules and ICVs at baseline and activation to find the best fit.

### 2.4 The effect of depth‐dependent vs constant CBV on the simulated profiles

**FIGURE 3:**
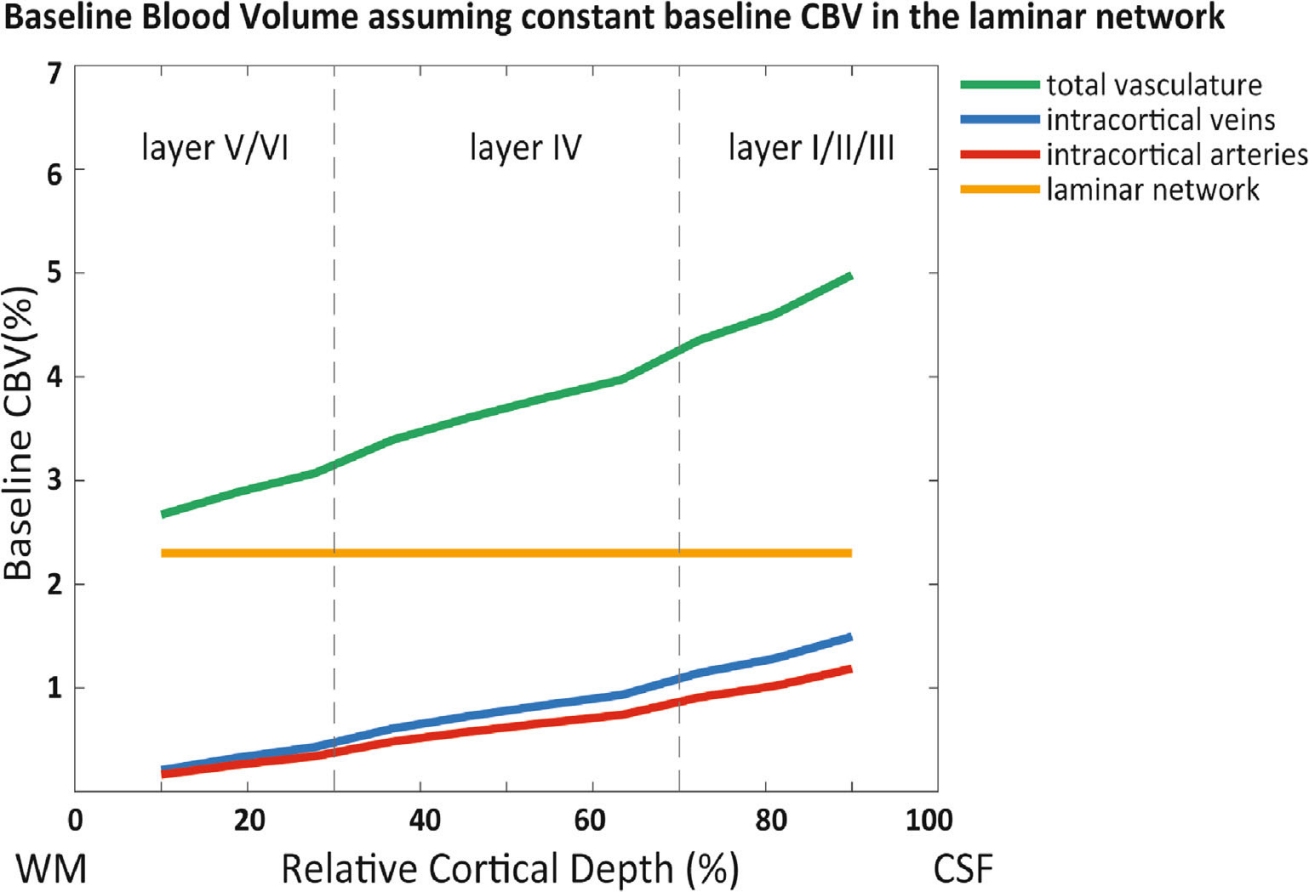
Baseline blood volume of the laminar network (i.e., arterioles, capillaries, venules), intracortical arteries, veins, and total vasculature in the “constant” baseline CBV scenario. We used the average of the baseline CBV in the laminar network (2.3%) reported in Weber et al. (2008).

To investigate the effect of variation in baseline CBV across depths, we performed the same simulation and fitting procedures, but assumed a constant baseline CBV across depths in the laminar network. We used a baseline CBV value of 2.3% across layers, which is the average of the depth‐dependent baseline CBV (Weber et al., 2008, Figure 1b). Figure 3 shows the baseline CBV of the laminar network, the baseline CBV in ICVs and ICAs (model output), and the total CBV of the vasculature (average ~ 3.7%) for this scenario. The aim of these simulation was to investigate which impact the pattern of the baseline CBV (constant vs depth‐dependent) has on the simulated BOLD and VASO responses.

## 3 EXPERIMENTAL METHODS

### 3.1 Model implementation

The cortical vascular model was implemented in MATLAB (2018b, The MathWorks, Inc.). The code is available on gitlab (https://gitlab.com/AtenaAkbari/cortical-vascular-model) including the original version used in Markuerkiaga et al. (2016) (branch: originalCode), the implementation used for an earlier version of this work presented at the ISMRM 2020 in which intracortical arteries were not yet added (Akbari et al., 2020) (branch: vasoSignal), and the implementation used in this manuscript (branch: master).

### 3.2 Image acquisition

Imaging was performed on a 7 T whole‐body MR scanner (Siemens Healthcare, Erlangen, Germany), with a maximum gradient strength of 70 mT/m and a slew rate of 200 mT/m/s. A single‐channel Tx and 32‐channel Rx head coil array (Nova Medical, Wilmington, MA, USA) was used for radiofrequency transmission and signal reception. The slice‐selective slab‐inversion (SS‐SI) VASO sequence (Huber, Ivanov, et al., 2014) —derived from the earlier developments of VASO at 7 T(Hua et al., 2013)—was employed to scan 10 healthy participants (two females; age range 19–32 years) after giving written informed consent according to the approval of the institutional ethics committee. For each subject, BOLD and VASO images were acquired in an interleaved fashion in three runs with 400 volumes in each run (15 minutes total acquisition time per run). The sequence parameters were: volume TR=4.5s, TE=25ms, TI=1100ms, GRAPPA (Griswold et al., 2002) acceleration factor =3, isotropic voxel size =0.8mm, number of slices =26, partial Fourier in the phase encoding direction =6/8, in combination with a 3D EPI readout (Poser et al., 2010). The blood‐nulling time was chosen based on the assumed value of blood $T_{1}=2100\;\mathrm{ms}$ following earlier VASO studies at 7 T (Huber et al., 2015; Huber et al., 2016; Zhang et al., 2013). As this blood nulling time is shorter than the arterial blood arrival time, the expected inflow effect (flow of the noninverted fresh blood) is low (Huber, Ivanov, et al., 2014). In addition, to further reduce the inflow effect to the imaging slice, we used an inversion pulse phase skip enabled in the sequence task card (Huber, Ivanov, et al., 2014). Fat suppression was also applied in all imaging protocols. Nevertheless, due to the short $T_{2}$ of the fat components, most of the fat signal decays during the EPI readout. In addition, since the residual fat artifact is expected to be the same at all time points, its effect on the functional signal can be considered negligible (Huber et al., 2016).

The visual stimulus consisted of 17 ON‐ and OFF‐blocks with 30 s duration each. During the ON condition, a flashing black and white noise pattern was presented, and a fixation cross was the OFF condition of the stimulus (Polimeni et al., 2005). The imaging slices were positioned and oriented such that the center of the imaging slab was aligned with the center of the calcarine sulcus, the part of the striate cortex with the highest vascular density in layer IV (Duvernoy et al., 1981). Whole‐brain MP2RAGE images (Marques et al., 2010; O'Brien et al., 2014) were acquired with an isotropic resolution of 0.75 mm for each participant in the same session as the functional imaging.

### 3.3 Image analysis

The first volume of each contrast was discarded to ensure $T_{1}$ effects were at equilibrium. Dynamic division was then performed to account for the BOLD‐contamination (Huber, Ivanov, et al., 2014). The BOLD and BOLD‐corrected VASO images were motion corrected using SPM12 (Wellcome Department, UK). Activation maps were estimated with the GLM analysis in SPM with no spatial smoothing. Data from three participants were discarded due to excessive motion, that is, volume‐to‐volume displacement of more than one voxel size. Voxels with t‐values above 2.3 corresponding to an uncorrected significance level of p<.01 were identified as the activated regions for both BOLD and BOLD‐corrected VASO images.

**FIGURE 4:**
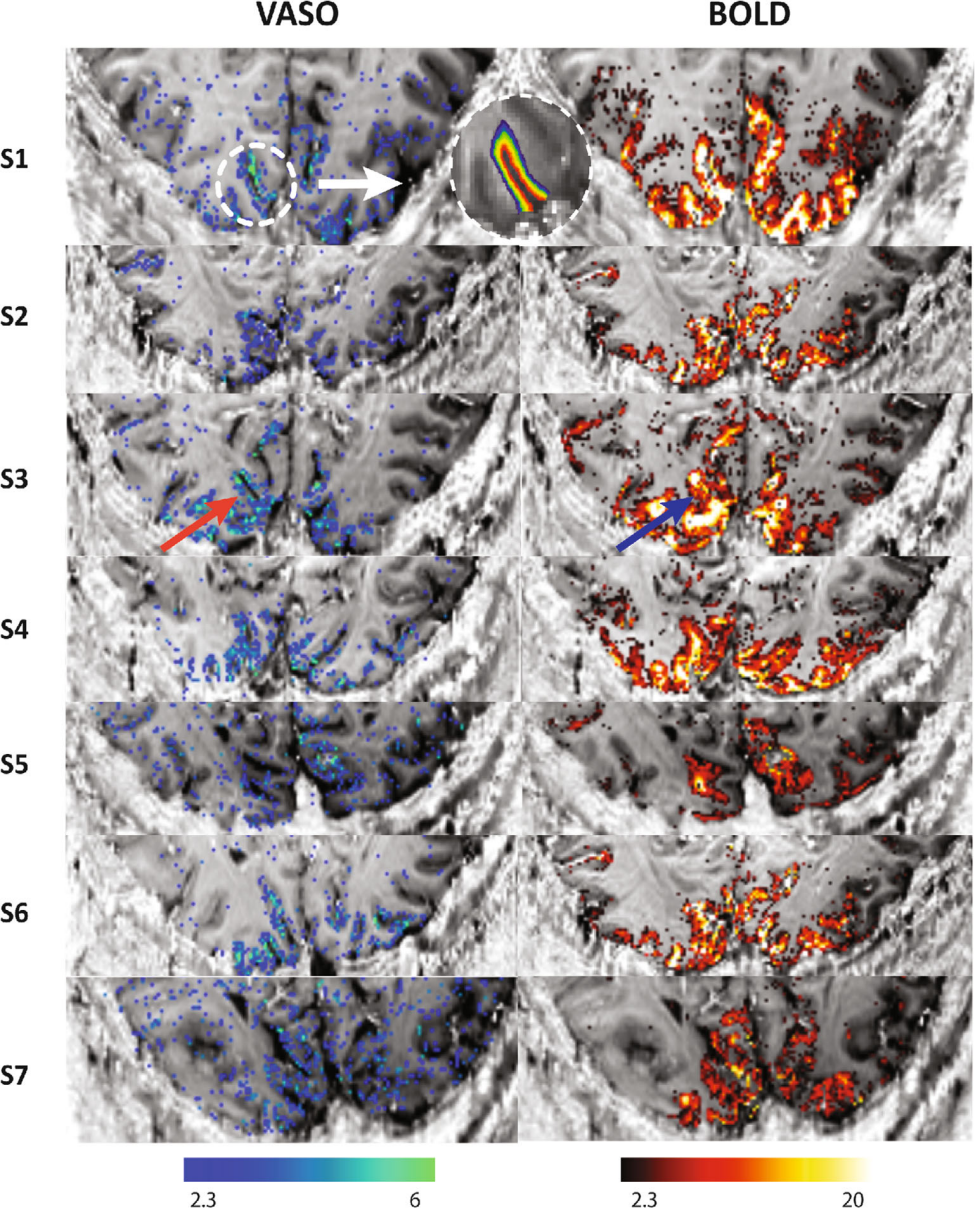
VASO and BOLD statistical activation maps of all participants in our study using the SS‐SI VASO sequence (Huber, Ivanov, et al., 2014) with an isotropic resolution of 0.8 mm. The activation maps are overlaid on T1‐EPI images of each subject. The VASO contrast is more confined to GM while BOLD shows higher activity near the surface (indicated with the red and blue arrows). An example of the region‐of‐interest (ROI) in V1 for the layer analysis is shown above. Ten equi‐volume layers were extracted from GM in the T1‐EPI images to calculate the mean signal change in each layer. No grey matter mask was applied to display the BOLD and VASO activation maps.

For the layer analysis, we followed the steps outlined in Huber, Ivanov, et al. (2014): The $T_{1}-\mathrm{EPI}$ images of each subject were used for WM/GM and GM/CSF boundary delineation, and a region of interest (ROI) was manually defined such that the activated regions in the calcarine sulcus from both contrasts were included (see Figure 4). Then, this ROI was used to create 10 equi‐volume layers (Waehnert et al., 2014) using the open‐source LAYNII package (Huber et al., 2021) and extract depth‐dependent BOLD and VASO responses. The average of the percentage signal change extracted from each layer forms the layer profile in both VASO and BOLD contrast. The mean and standard error of the mean were calculated across participants, and average BOLD and VASO responses across the seven participants were used as a reference when evaluating the model predictions. Note that cortical layers in these analyses refer to a group of voxels obtained by dividing the ROI into 10 equi‐volume layers and do not refer to the histological cortical layers. In the next section, we will first present the imaging results, and then introduce the simulations that fit these best. Further, we will present the simulated profiles with an RMSE 20% higher than the minimum RMSE, and the ΔCBV and oxygenation values corresponding to these profiles.

## 4 RESULTS

### 4.1 Imaging

The BOLD and VASO activation maps of all seven participants included in this study are shown in Figure 4. We observed overall higher t‐values for the BOLD contrast compared to the VASO contrast. Further, highest t‐values for BOLD are located at the cortical surface and within various sulci. In contrast, most of the VASO response is confined to the grey matter. An example of the ROI placed on V1 to extract the 10 equi‐volume layers and to estimate the depth‐dependent profiles, is shown for one subject in Figure 4.

**FIGURE 5:**
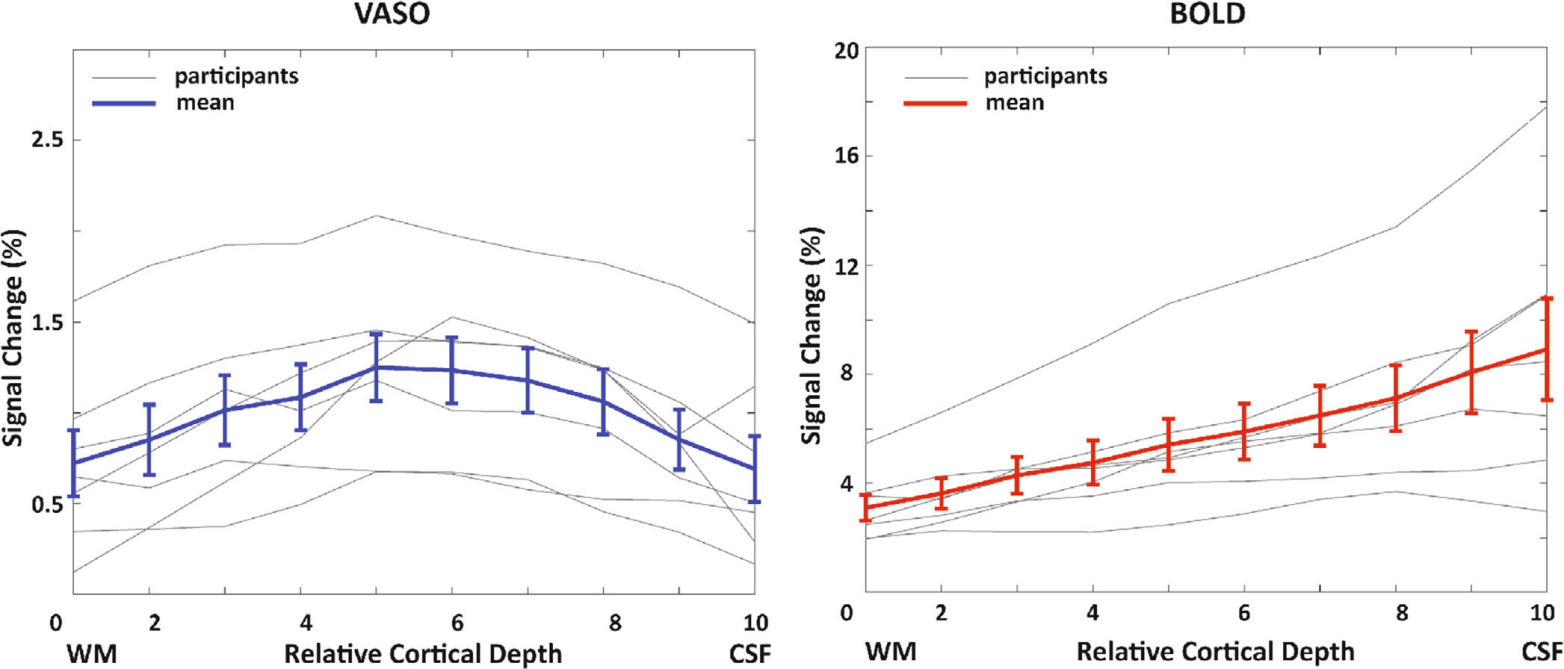
Depth‐dependent VASO and BOLD signal changes (%) in human V1 for each individual participant (gray) and averaged across all participants (blue and red). Note that the profiles plotted here are the average of the percentage signal change extracted from each layer. The error bars in this and all following graphs refer to the standard error of the mean across all participants.

The depth‐dependent BOLD and VASO signal changes for each participant as well as the mean and standard error of the mean of these profiles are shown in Figure 5. On average, we observed a mean signal change of 6% for BOLD and 1% for VASO, evidence for the larger effect size of the BOLD contrast. In agreement with previous studies, BOLD signal change peaks at the cortical surface (Koopmans et al., 2010; Olman et al., 2012; Polimeni et al., 2010) while the VASO signal change has its maximum in the middle cortical layers (Huber, Goense, et al., 2014).

### 4.2 Simulations

#### 4.2.1 Depth‐dependent BOLD and VASO profiles

**FIGURE 6:**
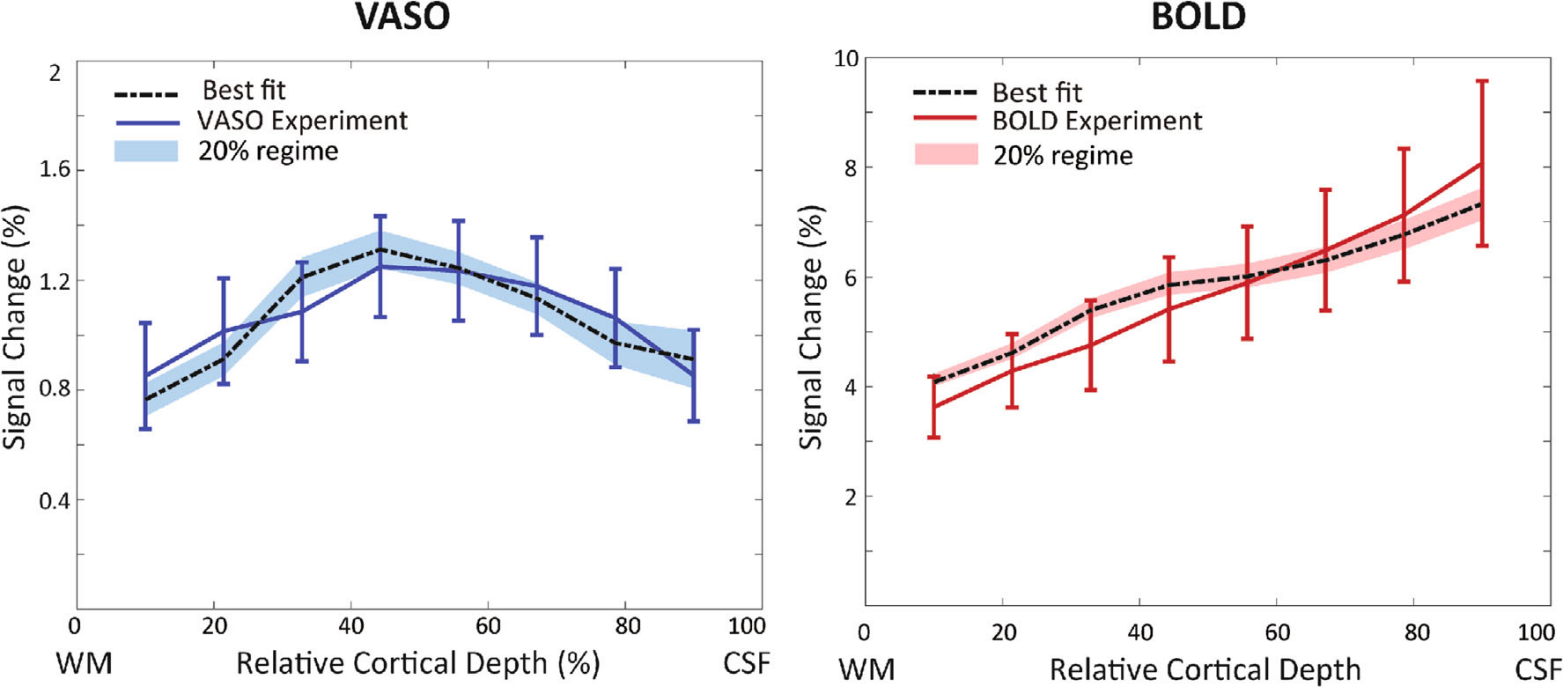
The measured VASO (left) and BOLD (right) profiles and the simulated profiles with the lowest RMSE (black line) assuming a depth‐dependent baseline CBV in the laminar network (Figure 1). The shaded area shows the VASO and BOLD simulated profiles with RMSE 20% higher than the minimum RMSE.

The simulated VASO profile with the best fit using a depth‐dependent baseline CBV in the laminar network (Figure 1) is shown in Figure 6, and corresponding CBV changes are shown in Table 4. We estimated highest CBV changes in arterioles and capillaries (56%) and in ICAs (21%), and smaller CBV changes in venules (2%) and ICVs (5%). For BOLD, the simulation with the best‐fit yields $\;Y_{\text{base}}=70\%$ and $\;Y_{\mathrm{act}}=90\%$ in venules and ICVs. To investigate the sensitivity of the model to the choice of input parameters, the shaded area in Figure 6 illustrates the range of profiles with an RMSE up to 20% higher than the minimum RMSE. The resulting profiles remain predominantly within the standard error of the measured profiles.

**TABLE 4:** CBV changes in vascular compartments corresponding to the best‐fit shown in Figure 6, that is, assuming a depth‐dependent baseline CBV in the laminar network.

CBV change in vascular compartments				
Cortical depth	Arterioles and capillaries	Venules	ICAs	ICVs
Deep (V, VI)	37%	1%	14%	3%
	[21% 41%]	(0% 49%]	[0% 42%]	[0% 42%]
Middle (IV)	56%	2%	21%	5%
	[32% 62%]	[0% 73%]	[0% 63%]	[0% 63%]
Superficial (I, II/III)	37%	1%	21%	5%
	[32% 62%]	[0% 49%]	[0% 63%]	[0% 63%]

*Note*: The minimum and maximum of the estimated CBV change in the 20% RMSE regime (i.e., the minimum RMSE +20% of the minimum RMSE shown as the shaded area in Figure 6) are shown in brackets.

#### 4.2.2 The effect of constant baseline CBV on the simulated profiles

**FIGURE 7:**
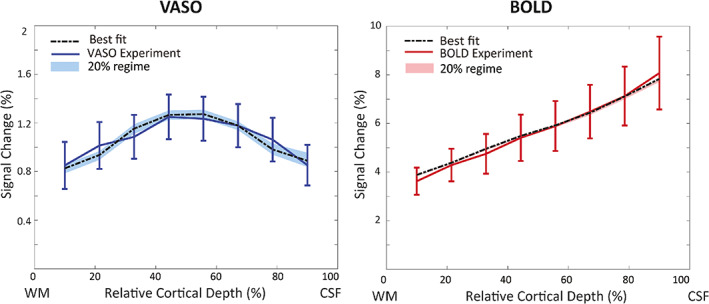
The measured and simulated VASO and BOLD profiles assuming a constant baseline CBV of 2.3% in the laminar network. The shaded areas show the RMSEs that are 20% higher than the minimum RMSE. The high agreement between BOLD simulation and measurement resulted in a low minimum RMSE and a correspondingly very narrow 20% RMSE regime.

Figure 7 illustrates the simulation results assuming a constant baseline CBV across depths in the laminar network (Figure 3). This assumption produces a better fit for both VASO and BOLD profiles and a smaller 20%‐RMSE regime (shaded area). Note, however, that in the laminar network we are still assuming a 1.5 times higher CBV change in middle layers than in superficial and deep layers (Figure 2, Equation 14). Again, highest CBV change was estimated in arterioles and capillaries (65% in middle layers), and small CBV changes in all other compartments (Table 5). For BOLD, the best fit estimates 71% and 90% oxygen saturation at baseline and activation, respectively.

**TABLE 5:** CBV changes in vascular compartments corresponding to the best fit shown in Figure 7, that is, assuming a constant baseline CBV of 2.3% in the laminar network.

CBV change in vascular compartments				
Cortical depth	Arterioles and capillaries	Venules	ICAs	ICVs
Deep (V, VI)	43%	0%	2%	1%
	[28% 45%]	[0% 49%]	[0% 10%]	[0% 10%]
Middle (IV)	65%	0%	3%	2%
	[42% 68%]	[0% 73%]	[0% 15%]	[0% 15%]
Superficial (I, II/III)	43%	0%	3%	2%
	[28% 45%]	[0% 49%]	[0% 15%]	[0% 15%]

*Note*: The minimum and maximum of the estimated CBV change in the 20% RMSE regime (i.e., the minimum RMSE+20% of the minimum RMSE shown as the shaded area in Figure 7) are shown in brackets.

#### 4.2.3 The effect of equal activation strength across depths

**FIGURE 8:**
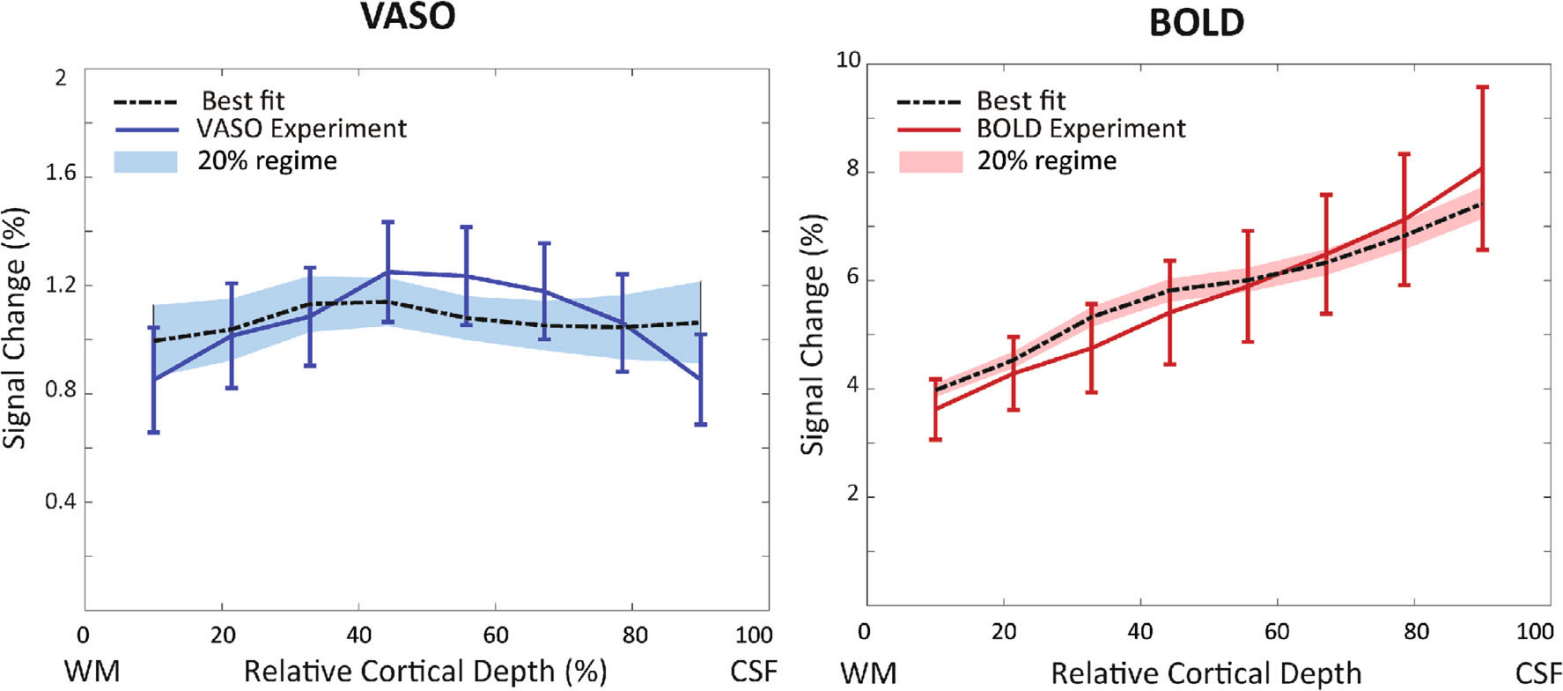
The measured and simulated VASO and BOLD profiles assuming a depth‐dependent baseline CBV in the laminar network, but constant activation strength across depths. The simulated VASO profile deviates considerably from the measured response, whereas the fit to the BOLD data is comparable to the scenario with depth‐dependent nonuniform activation strength.

The simulation results of equal activation strength across layers (i.e., β=1 for all layers in Equation 14 such that all layers are activated equally) are shown in Figure 8. Note that this equal activation strength across depths was the assumption in the original implementation (Markuerkiaga et al., 2016). However, under this assumption, the VASO profile is much flatter and does not show the expected higher CBV change in middle layers (Goense et al., 2012; Poplawsky & Kim, 2014; Zhao et al., 2006). In contrast, this scenario had little impact on the predicted BOLD profile that showed comparable characteristics and similar fitting performance, but the experimental VASO profile could not be reproduced. Using a depth‐dependent baseline CBV, the best fit estimates 48% CBV change in arterioles and capillaries, 3% in venules, 12% in ICAs, and 9% in ICVs in middle layers. The corresponding oxygenation levels at baseline and activation are 65% and 86%, respectively.

## 5 DISCUSSION AND CONCLUSION

In this study, we extended and modified the cortical vascular model (Markuerkiaga et al., 2016) to simulate depth‐dependent VASO signal changes in addition to BOLD signal changes and added intracortical arteries to the modelled area for a full description of CBV changes in the intracortical vasculature. With our simulations, we found that stimulus evoked CBV changes are dominant in small arterioles and capillaries at 56%, and in ICAs at 21%, and that the contribution of venules and ICVs is smaller at 2% and 5%, respectively. These estimates of higher arterial CBV change are in line with previous studies, for example, in mice with 60 s stimulus duration (Takano et al., 2006), cats with 40 s stimulus duration (Kim & Kim, 2010, 2011b) and humans with 30 s or longer stimulus duration (Chen & Pike, 2009, 2010; Huber, Goense, et al., 2014). Chen and Pike (2009, 2010) showed larger venous CBV changes of ~10% in veins when using a mild hypercapnia (96 sec), and up to 20% venous CBV change with a moderate hypercapnia stimulus (180 sec). In a simulation study using the extended Windkessel model (Mandeville et al., 1999), Barrett et al. (2012) estimated dominant CBV change in arteries (64.8%) and smaller CBV change in veins (21.5%) with 30 s stimulus duration. In addition, it is also expected that the VASO contrast is overall less sensitive to CBV changes in venules and veins due to water exchange in the capillaries (Huber, Ivanov, et al., 2014; Jin & Kim, 2008b; Lu et al., 2003). Thus, overestimation of the arterial CBV change and/or underestimation of the venous CBV change is a possible outcome of these simulations due to the nature of the VASO contrast. In summary, the cortical vascular model allows to estimate and compare BOLD and VASO signal changes in various conditions and resolves the contributions of different vascular compartments to the fMRI signal.

The inclusion of ICAs allowed us to investigate the sensitivity of the VASO signal to upstream CBV changes. Although we found a relatively large CBV change of 21% in ICAs, the measured and simulated profiles did not show the pial bias that is commonly found in BOLD profiles (Barth & Norris, 2007; Kim et al., 1994; Turner, 2002). This might be due to the different contrast mechanisms of VASO and BOLD, where the VASO signal is linearly proportional to CBV (Equation 13). Together with the low baseline blood volume in ICAs (Figure 1C), even a relatively large CBV change in ICAs might thus only have a limited impact on the resulting VASO profile. In contrast, the extravascular signal contributions around venules and ICVs presumably amplify the effect of oxygenation changes in these vessels on the BOLD signal (Equation 7). Thus, the measured and simulated BOLD profiles are heavily skewed towards the signal stemming from ICVs. In conclusion, our results confirm that the VASO contrast is less susceptible to large vessel effects compared to BOLD (Huber, Ivanov, et al., 2014; Huber, Uludağ, & Möller, 2017; Lu et al., 2003; Lu et al., 2013).

In general, we found that a wide range of CBV changes in the different micro and macrovascular compartments can result in similar depth‐dependent profiles (Table 4); indicating potential challenges when aiming to invert the measured profiles. These variations are illustrated in more detail using RMSE plots (see Figures S3 and S4 in the Supplementary Materials), where we observed high differentiability between macro‐ and micro‐vascular compartments, but considerable interchangeability within each vascular class, such that for example a wide range of ΔCBV combinations in ICVs and ICAs can result in comparable VASO profiles, and thus comparable RMSE values. Similarly, a change in oxygenation of 20% provides the best fit for BOLD, but a wide range of oxygenation levels at baseline and a corresponding value during activity are possible

As the model fits the measured BOLD response well, we did not investigate the effect of the pial veins on the simulated laminar response. The absence of the pial veins in our simulation might also explain the residual mismatch between simulated and measured BOLD profile in Figure 7. In a recent study by Markuerkiaga et al. (2021) pial vessels were added to the model, while the contribution of downstream vasculature in the laminar GRE‐BOLD response was removed using a deconvolution approach. This resulted in more spatially specific laminar BOLD responses that no longer show the bias towards the cortical surface.

To investigate the influence of the assumed baseline CBV on the simulated profiles, we compared the effect of assuming either a depth‐dependent or a constant baseline CBV in the laminar network and found that a constant baseline CBV across depths provides a better fit to both BOLD and VASO profiles. However, this observation might be driven by uncertainties in defining an accurate depth‐dependent baseline CBV, namely (i) the baseline CBV used here was derived from ex vivo macaque data (Weber et al., 2008), (ii) the WM/GM and GM/CSF boundary definition was manually performed in EPI image space introducing potential inaccuracies, and (iii) the depth‐dependent profiles were averaged across participants. Consequently, an *average*, that is, constant baseline might perform better in this case.

The average of the total baseline CBV of the modelled vasculature is 3.7% (Figure 1D), in which 1.2% is the baseline CBV of ICAs, which is comparable to the values reported in Ito et al. (2001) (1.1 ± 0.4%), Ito et al. (2005) (1.5 ± 0.3), and Yan et al. (2012) (1.14–2.05). The baseline CBV of ICVs is 1.4%, lower than the values reported by He and Yablonskiy (2007) (1.75 ± 0.13) and Ito et al. (2005) (1.9 ± 0.5). For additional references see Tables 1 and 2 in Hua et al. (2019).

A slight divergence—but within the standard error of the mean—remains between simulated and measured data. Notably, the so‐called “bump” (Chen et al., 2013; Havlicek & Uludağ, 2020; Huber, Handwerker, et al., 2017; Koopmans et al., 2010) is visible in the simulated BOLD profile but not the imaging data. Preliminary investigations show that this feature seems to be mainly driven by the baseline CBV in this model (Figure 6 vs. Figure 7, see also Figure S1 in the Supplementary Materials), but in general is expected to have mixed neuronal and vascular origin (Havlicek & Uludağ, 2020). Note that this feature was also not evident in numerous studies using comparable acquisition and analysis techniques. In addition, the resolution of the imaging data (0.8 mm^3^) could be insufficient to detect this bump in the laminar BOLD profile in the thin human visual cortex, and the use of an EPI readout might also introduce blurring and partial voluming that smooth out this local maximum in the laminar BOLD profile (Huber et al., 2018). In a study by Fracasso et al. (2018), in addition to a group of laminar BOLD profiles demonstrating a monotonic increase towards the surface, a group of laminar BOLD profiles exhibited a peak at the middle cortical layer. They argued that this peak might (i) reflect the signal originating from the micro vessels in input layer of V1; (ii) depend on the extent to which a pial vein was included in a profile, (iii) be simply due to the realignment error in the registration process; (iv) be due to the local distortion in the EPI space.

The various parameters used to build the cortical vascular model such as blood velocity, vessel diameter, and baseline blood volume in capillaries were taken from previous research in rats, cats, rabbits, and macaques (Markuerkiaga et al., 2016; Weber et al., 2008; Zweifach & Lipowsky, 1977), which might impose uncertainties on the estimated CBV changes and simulated profiles. However, we took into account the inter‐species differences in vascular structure and density by reversing the arterioles‐to‐venules ratio defined in the VAN model (Boas et al., 2008), as the arterial density in the human brain is higher than its venous density in contrast to the ratio in the rat brain (Baez‐Yanez et al., 2020;Cassot et al., 2009; Schmid et al., 2019). Here, we assumed an artery‐to‐vein ratio of 2‐to‐1 (Cassot et al., 2009; Schmid et al., 2019), but larger artery‐to‐vein ratios of 2.58 (Adams et al., 2015) and smaller artery‐to‐vein ratios of 1.6 (Weber et al., 2008) have also been reported in the literature for macaque brain. The simulation results following from the assumption of the original cortical vascular model (Markuerkiaga et al., 2016) of an arterioles‐to‐venules‐ratio of 1‐to‐2 in the laminar network (as reported in the VAN model for the rat brain) are shown in the supplementary materials (Figure S9). This assumption failed to explain our laminar BOLD imaging data by demonstrating a relatively large discrepancy between simulated and measured profiles. Reversing the arterioles‐to‐venules ratio improved the fit to the BOLD profile noticeably (compare Figures 6 and S9). Such dependence on the assumption of the vascular network composition suggest that care must be taken when using the reported values from the rat brain for human brain simulations. Further, we noticed that for certain parameter combinations the derived vessel diameters and blood velocities in the ICAs and ICVs can easily contradict previous reports that intracortical arteries have smaller diameter (Duvernoy et al., 1981) and faster blood velocities (Zweifach & Lipowsky, 1977). Thus, while the cortical vascular model aims for a detailed description of the underlying micro and macrovasculature and its influence on the MR signal, many uncertainties in the specific parameter choices remain. One example includes the dilation profile of the ICAs across cortical depths, where we assumed a higher vascular response in middle and superficial layers (Figure 2). However, another possible scenario could be an equal activation strength in ICAs across cortical depths (see Figure S5 and Figure S6 of the Supplementary Materials), which results in similar depth‐dependent VASO and BOLD profiles, but different estimated CBV changes. Additionally, the inter‐individual variability in these parameters remains unknown, but may potentially have a large effect on the individual profiles given the many studies showing significant differences in hemodynamic responses between participants (Aguirre et al., 1998; Duann et al., 2002; Handwerker et al., 2004; Light et al., 1993). Consequently, a more detailed understanding of the relative impact of each of these parameters needs to be developed, in combination with auxiliary image acquisitions that measure relevant underlying parameters.

The experimental results show similar profiles as expected from previous research (Huber et al., 2013; Huber et al., 2016; Jin & Kim, 2006, 2008b; Koopmans et al., 2010). To ensure highest contrast‐to‐noise ratio when comparing with the simulations, we have averaged the responses across participants. We extracted percent signal change values using a GLM, assuming the same hemodynamic response for all cortical layers. Although each layer has a unique HRF (Petridou & Siero, 2017), we expect a negligible bias in the estimated signal change due to the very long stimulus time employed here. There is also evidence of the dependency of blood $T_{1}$ on hematocrit levels (Dobre et al., 2007) affecting the blood nulling time, though the effect can be considered negligible.

The vascular anatomical model used here presents a simplification of vascular anatomical networks (VAN) (Boas et al., 2008; Gagnon et al., 2015; Genois et al., 2020), but employs more details in the mirco and macrovasculature than the fully invertible model developed by Havlicek and Uludağ (2020). Thus, it is uniquely suited to translate new insights from detailed VAN models developed in rat to the dynamic laminar models used to fit human data. As exemplified in this work using changes in CBV, the impact of each parameter on the resulting laminar profiles can be assessed individually, to then inform the choice of acquisition, potential vascular biases, and the need for auxiliary information. Next, the vascular anatomical model can be extended to other cortical areas characterized by different vascular properties such as primary motor cortex (Huber, Handwerker, et al., 2017), primary somatosensory cortex (Oliveira et al., 2021; Shih et al., 2013; Silva & Koretsky, 2002), dorsolateral‐prefrontal cortex (Finn et al., 2019), which are currently under active investigation using laminar fMRI to help to understand the vascular and neural signal contributions. In addition, the potential of higher spatial resolution can be explored with the model. For example, inspired by the study of Huber et al. (2015) who measured CBV responses in the macaque brain at 500 μm resolution and observed a double‐peak pattern, that is, local maxima on both sides of the stria of Gennari, we simulated a depth‐dependent VASO profile but without applying any smoothing kernel. Interestingly, the model prediction indeed features a pattern that resembles a double peak corresponding to higher local CBV changes on both sides of stria of Gennari (Huber et al., 2015) (see Figure S7 in the Supplementary Materials).

In summary, we acquired BOLD and VASO laminar responses in human V1 at 7 T and simulated these responses using the cortical vascular model. To the best of our knowledge, this is the first study to acquire the laminar BOLD and VASO profiles in addition to simulating these responses in the human primary visual cortex. By fitting the model to our experimental results, we obtained an estimate of CBV change in all vascular compartments upon neural activity. Our simulation results show that stimulus evoked CBV change is dominant in small arterioles and capillaries followed by ICAs, and the contribution of venules and ICVs in total CBV change is small when the stimulus is relatively long (~30 sec). Our results also suggest that the large vessel bias is less prominent in VASO contrast compared with BOLD, as the BOLD signal relationship with the oxygenation change is exponential, but VASO depends on the CBV change linearly.

## CONFLICT OF INTEREST

The authors declares there is no potential conflict of interest.

## Supporting information


**Appendix S1** Supplementary Information

## Data Availability

The measured BOLD and VASO profiles and the MATLAB code are publicly available via Gitlab.

## Footnotes

^1^
Note that $S_{\mathrm{EV}}^{\text{non}-\text{nulled}}$ corresponds to the extravascular BOLD signal. During the BOLD correction of the experimental data, we assume the intravascular BOLD signal to be negligible following Huber et al. (2014), and Genois et al. (2020).
